# Integrating competency-based education with a case-based or problem-based learning approach in online health sciences

**DOI:** 10.1007/s12564-020-09658-6

**Published:** 2020-11-18

**Authors:** Ilse Johanna Sistermans

**Affiliations:** Maastricht University, Maastricht, The Netherlands

**Keywords:** Online learning, Competency-based education, Problem-based learning, Case-based learning, Online learning activities, Health sciences

## Abstract

In the current competitive and globalized economy, employers and professional organizations call for higher education institutions to deliver graduates with relevant competencies and skills. In response, a growing number of higher educational institutions is introducing competency-based education. This is particularly true for health science programs, which have a tradition of applying a case-based or problem-based learning approach. The effort to merge a problem- or case-based online learning approach with competency-based education offers various opportunities, while facing numerous challenges. To support these efforts, this paper aims at identifying suitable practices, as well as challenges for online course design and online learning activities for higher education health science programs, when integrating competency-based education with an online problem-based and/or case-based learning approach. It found various opportunities for online learning activities that support competency-based education, problem-based learning and case-based learning, whereas challenges relate to logistics, administration, and the affordances of an LMS.

**Electronic supplementary material:**

The online version of this article (10.1007/s12564-020-09658-6) contains supplementary material, which is available to authorized users.

## Introduction

In response to the current competitive and globalized economy, there is a call from employers and professional organizations for higher education institutions to deliver graduates who possess relevant competencies and skills. A growing number of educational institutions and workplace training programs have therefore introduced competency-based education, or are planning to do so, with a significant number of higher education health science programs applying constructivist approaches of problem-based and/or case-based learning. Competencies vital to a specific professional field have been formulated by corresponding national and international professional organizations. Examples are the frameworks by the International Union for Health Promotion and Education (IUHPE), and the United States National Board of Medical Examiners (Gruppen et al. 2012).

Maastricht University Master Health Education and Promotion (HEP), is seeking to restructure the program toward a competency-based approach, in order to comply with the framework, recently developed by the International Union for Health Promotion and Education (IUHPE), that addresses competencies required for graduates seeking a career in the field of health promotion and education (Barry et al. 2012). In addition, the program is making its first steps in moving from a face-to-face problem-based program, toward a (partly) online program with problem-based and case-based elements. However, the availability of online modules within the program, relies on institutional funding.

Integrating competency-based elements into an existing time-based curriculum that caters to cohorts, is a complicated, costly intervention. Moreover, guiding and providing support for a personal, self-paced learning paths can be a challenge to the logistics of a program, as are modes of assessment (Porter and Reilly 2014).

The purpose of this paper is to identify suitable practices, as well as challenges, for online course design and online learning activities for higher education health science programs, when integrating competency-based education with an online problem-based and/or case-based learning approach. In addition, it tries to bring clarity to the professional conversation by defining and analyzing (online) problem- and case-based learning, as well as (online) competency-based education, in an effort to inform and inspire teaching staff and program coordinators and directors, particularly of tertiary health science education programs, who intend to transfer from a face-to-face to an online environment. To that end, interviews conducted with higher education health professionals in the Netherlands, Belgium and the United States of America were combined with a literature review.

## Literature review

### A brief history of face-to-face, distance and online education theory

#### From teacher-centered to learner-centered approaches

The theories and related pedagogies behind face-to-face and distance education have evolved over the last century. Pedagogy of education is based on theories that relate to knowledge, its location, how it is transmitted, retained and/or constructed. The earliest learning theory, behaviorism, focuses on human behaviors rather than processes within the mind. The behaviorist learning process is based on repetition and learner response to stimuli (Anderson 2017), and now learning can be observed and quantified. Cognitivism followed behaviorism after researchers started experiencing limitations in the ability of behaviorism to describe learning process (Harasim^2012^). Cognitivist theorists see learning as an internal process of the mind that takes place between knowledge input and output – similar to computer information processing (Jordan et al. 2008). The input consists of “material from memory” or “sensory data”; the learning process involves “attention”, “perception”, “encoding”, and “memory”; the output includes “action, “retrieval”, and “storage in long-term memory” (Jordan et al. 2008, p. 37). In education, behaviorism and cognitivism tend to have a teacher-centered approach, where the teacher sends information to the learner(s) (Harasim 2012).

In the seventies, constructivism was introduced as a learning theory and epistemology that poses a learner constructs his or her knowledge based on their own experience, with an understanding of their environment coupled with reflection on that experience (Harasim 2012). Constructivism distinguishes itself from behaviorism and cognitivism, as it is learner-centered. Additional key elements of the constructivist learning approach are its collaborative, active, and reflective nature, and relevance to the students, who is encouraged to act autonomously and self-directed (Lebow 1993). Two constructivist learning approaches this paper investigates, are problem-based learning (PBL) and case-based learning (CBL).

#### From distance education to online learning

Behaviorism, cognitivism and constructivism all have informed distance education practices, over the course of time. The separating factor between distance education and face-to-face education has been the physical distance between teacher and learners that distance education needs to overcome. Media required to bridge that geographical distance, have strongly influenced distance education pedagogy throughout its history and linked it to technological innovation (Cleveland-Innes and Garrison 2010). Distance education started as correspondence learning, where the medium was printed learning material coupled with the form of transport required to exchange learning material, assignments, corrected work, evaluations and feedback between students and teachers. Inventions, such as telephone, radio, TV, video, DVD, computers, the World Wide Web (Holmberg 2005), virtual learning environments, virtual reality, artificial intelligence, smartphones, apps and other communication and information technology developments, have impacted how learners interact with their teachers, peers, and learning material, and, subsequently, the distance (and later online), teaching and learning approach and underpinning theories.

Recent changes in communication technologies have been accompanied by the emergence of new distance education theories relating to interaction. This includes the “didactic conversation”, later renamed “empathy approach” by Holmberg (2005, p. 3), on an empathetic friendly didactic approach to writing. Next theory is the transactional distance theory by Micheal Moore, which claims, “distance is a function of structure and dialogue” (Bernath and Vidal 2007). Furthermore, the interaction equivalency theory relates to interaction between “student–teacher”, “student-student”, and “student-content” (Anderson 2003, p. 3). Finally, the Community of Inquiry (CoI), a theoretical framework by Garrison, Anderson, and Archer (2000) is relevant within this context; it relates to teaching presence, cognitive presence and social presence in online learning.

Along with the affordances of online technologies and introduction of Web 2.0 and Web 3.0, and web conferencing tools, distance education grew increasingly interactive. Whereas previously, communication had been asynchronous, online learning affords both an asynchronous and a synchronous mode of communication between teacher and students and among students (Harasim 2012). The decision to use either mode of communication can now be informed by pedagogical factors, rather than media limitations.

Theorists have argued whether online education is merely a new variation of distance education, or a new form of education altogether (Anderson 2009; Garrison 2009). Whoever is right, it is safe to conclude that the affordances of online interaction have brought face-to-face and online learning closer than ever before, in terms of didactic approach. However, it is important to realize that even with the same theoretical underpinnings, face-to-face and online learning continue to require a different approach to course design and learning activities.

### Comparing problem-based and case-based learning

Problem-based learning is a constructivist experiential, student-centered approach to learning that facilitates the integration of theory and practice, and the application of knowledge and skills, enabling learners to reach a viable solution to an ill-defined, real-world (authentic) problem (Savery 2015; Ten Cate 2007). It involves a self-directed, constructive learning process (Ertmer and Newby 2010; Moust et al. 2013) that promotes student competencies such as collaboration, evaluation, and critical and analytical thinking skills, and enables students to solve problems in various contexts (Savery 2015). Problem-based learning (PBL) was first implemented in medical education at the Canadian McMaster University, during the late sixties (Savery 2015; Ten Cate 2007). Its origins have also been attributed to Case Western Reserve University School of Medicine, twenty years earlier. Over the years, PBL was adopted by health sciences and other disciplines in higher education (Blumhof et al. 2001).

The focal point of problem-based learning is not the answer to the problem itself, but the process toward finding the solution and the skills and competencies that students build during this process. Students define and analyze a problem statement, and define their own learning goals, evaluate information resources, establish and test hypotheses, integrate concepts and come up with, and present valid solutions to others, and receive and integrate (teacher or peer) feedback (Blumhof et al. 2001; Moust et al. 2013). At Maastricht University, where a problem-based curriculum has been implemented for most tracks, the first phase of the problem-solving process is referred to as ‘pre-discussion’, and is often followed by individual research, i.e., self-study. The cycle is finalized by a ‘post-discussion’ (Moust et al. 2013).

The first application of case-based learning (CBL) is attributed to a pathology professor at the University of Edinburgh, in 1912 (Sturdy 2007, as cited in Thistlethwaite et al. 2012). CBL is also described as “case study teaching and case method learning” (Thistlethwaite et al. 2012, p. 421), and is a form of constructivist experiential, self-regulated learning, aimed at activating learners and promoting higher order thinking skills, i.e., analyses and synthesis and critical thinking skills, problem-solving skills, self-reflection, and empathy. Learners work through a well-constructed authentic case, based on a real case and using real quotes, aligned with the intended learning outcomes (Thistlethwaite et al. 2012). They are encouraged to discover and fill knowledge gaps needed to solve the case (Nicklen et al. 2016). The aim is to build knowledge that can be applied to similar situations in the future, and, if conducted as a group, case studies stimulate authentic professional collaboration (Lyons and Bandura 2017; Savery 2015).

Both case-based and problem-based learning are learner-centered constructivist learning approaches in which core principles are collaboration, self-regulation, and promoting higher order thinking skills that students can develop through activities such as feedback, discussion and (self) reflection (Savery 2015; Ten Cate 2007; Thistlethwaite et al. 2012). Both have been developed in the medical domain where collaboration, interdisciplinary teams and analytical skills, problem-solving skills, critical thinking skills, reflective skills and empathy were important competencies for graduates. While case-based learning often departs from a well-defined case, problem-based learning departs from an ill structured problem and requires students to formulate their own problem statements to be solved (Savery 2015; Ten Cate 2007).

Not all reviewed literature regard problem-based and case-based learning as two distinctive learning approaches; for instance, Cheaney and Ingebritsen assert that the two approaches are the same; they describe problem-based learning as learning that involves the use of “a real-world problem or situation as a context for learning” (2006, para. 2). Furthermore, they describe common constructivist elements of PBL and CBL, such as the ‘development of critical thinking skills”, “problem-solving abilities” and “self-directedness” important for “life-long-learning” (Cheaney and Ingebritsen 2006, para. 2).

### Online problem-based and case-based learning

The introduction of web 2.0 and 3.0 tools facilitates constructivist learning approaches, such as problem- and case-based learning. An increasingly “interactive” and “non-sequential online environment” fits problem-solving process of a group of learners (Wilkie and Savin-Baden 2006, p. 10). Online collaborative learning is a constructivist online learning approach that focuses on knowledge construction, skill building, and problem-solving through discussion and collaborative assignments as well as webinars (Harasim 2012). Generally, online CBL or PBL curricula design is informed by online collaborative learning theory.

Critics, however, argue that an online environment negatively affects the group process that is central to problem-based learning. In their opinion, it would narrow the scope of the problem, thus limiting the self-directedness of learning (Wilkie and Savin-Baden 2006).

### Competency-based education

Competency-based education (CBE) finds its origins in outcome-based education in the late forties, with educational psychologists Ralf Tyler as one of its first theorists. CBE became a more common approach in medical education around the turn of the millennium (Ten Cate 2017). In the recent past, the Korean Accreditation Board of Nursing Education (KABONE), went as far as making outcome-based learning mandatory and formulated seven core competencies (Lee et al. 2018). In the EU, the implementation of competency-based and competency-oriented education is supported by the Bologna process, which also introduced the bachelor-master cycle, a transferable credit system (ECTS) to European higher education, and promotes learner mobility and life-long learning.

A significant part of the reviewed literature employs the terms competency-based and outcome-based learning interchangeably. Furthermore, there are several definitions of competency-based education, but most include at least four characterizations. First, learners need to prove mastery of a skill or competency for (micro) credit and/or to advance to a next level. Second, learners can obtain extra time and/or personal instruction. Third, the assessment of student’s mastery takes place through skills application. Fourth and final, a traditional classroom setting is not a prerequisite, as learning can also take place on the work floor, during an internship, job shadowing, through online, blended or distance learning, or otherwise (Scheopner-Torres et al. 2015).

The author found various definitions of a competence or competency. One definition of a competence is an individual’s “ability to act within a given context in a responsible and adequate way, while integrating complex knowledge, skills and attitudes” (Van der Blij, as cited in Ehlers et al. 2008). The Tuning project formulated an alternative definition of competencies or competences: “a combination of cognitive and metacognitive skills, demonstration of knowledge and understanding, interpersonal, intellectual and practical skills and ethical values” (as cited in Zelvys and Akzoholova 2016, p. 187).

Like problem-based and case-based learning, competency-based education is linked with student-centeredness and self-directedness. In CBE, learning can take place in a (face-to-face or online) classroom setting, as well as outside the classroom. The assessment commonly includes feedback by peers as well as self-assessment. Learning or developing a competency can be broken into smaller steps. These steps have been described in frameworks, such as the Dutch competency standard framework that describes a five increasingly higher competency levels, from 1 “basic knowledge and basic professional behavior” to 5, “independently perform the professional activity” (Skowron et al.^2017^). Within the field of medicine, various international competency frameworks were introduced, such as the Canadian CanMED model and the US ACGME system. These frameworks cut the end terms into “entrustable professional activities” (EPAs); each EPA demonstrates the level of proficiency of a medical professional (Ten Cate 2013, p. 157).

### Online competency-based education

Reviewed literature, describes competency-based education (CBE) as a student-centered, self-directed and experiential approach, facilitating skill and competency development, including higher order thinking skills and problem-solving skills (Ehlers et al. 2008; Tan et al. 2018). D. L. Anderson defines competency-based education, as follows: “CBE offers a flexible way for students to earn credit based on demonstration of subject-matter knowledge earned either through self-paced instruction or examination based on mastery of competencies” (2017). The use of the terms “subject-matter” and “self-paced,” leads some to conclude that automated self-paced online instruction (without a tutor) is a form of competency-based education. In case of competencies in health science students and many other learners, however, such an online self-paced module is limited in the extent to which it can foster experiential learning, and interpersonal skills development.

## Methods

This qualitative research aims to identify best practices and challenges for online course design and suitable learning activities for a higher education health science program, when integrating problem-based or case-based learning with competency-based education.

The main instrument of this research is a standardized open-ended interview conducted with six higher education professionals in the field of online Health Sciences and Health Science Education. A standardized open-ended interview is suitable for the small number of interview participants. While open-ended questions allow for more in-depth responses, the structuring “facilitates data structuring, comparison and analyses” (Cohen et al. 2000, p. 271). The objective of the interview was to obtain a selection of online design and online learning activities that fit both an online problem-based and/or a case-based learning approach, and an online competency-based education approach, and to address opportunities and challenges.

The second instrument was a literature review. The goal of interview was to find a selection of examples of online curriculum design and online learning activities that fit both an online problem-based and/or case-based learning approach as well as online competency-based education. Additionally, it attempted to find opportunities and challenges encountered when merging online competency-based education with either problem-based or case-based learning through comparative analyses.

### Participants

This research was performed at the request of the program director of Maastricht University Master Health Education and Promotion (HEP), where the author is employed as a blended and online learning specialist. The HEP program director was seeking to restructure the program, which follows a problem and case-based learning principles, to a partly online format with a competency-based approach.

The participants were selected from the professional network of the HEP program director and author, based on their level of professional expertise, and their leading roles in online and blended higher education programs in the field of health sciences. The group of participants consists of a health sciences program coordinator, a health sciences department chair, a health sciences program director, an educational researcher at a faculty of medicine and health sciences, an e-learning program manager at a faculty of medicine, and a blended and online learning research coordinator at a faculty of education. Five are female and one is male within the age range of 30 and 61 years of age (M = 48), four participants hold a Dutch and one a Belgium nationality, and one is a US citizen. The selected six participants represent various programs at the following four institutes: Maastricht University (the Netherlands), Erasmus University of Rotterdam (the Netherlands), the Catholic University of Leuven (Belgium), and the University of Maryland University College (United States of America).

The interviews were held between July 16 and August 1, 2018; the three interviews with participants who were employed at Maastricht University took place in person and the other three interviews were conducted through video call. The interviewees did not receive any compensation. Dutch legal requirements for expert interviews do not require a human subject review committee, as experts are not subject to the Dutch Law on Research involving Human Subjects (WMO, http://wetten.overheid.nl/BWBR0009408/2017-03-01). The research did adhere to general ethical guidelines for interviews, seeking permission from the interviewees to record and quote their responses, and granting the interviewees the right to request confidentiality and the omission of any (part) of their responses, during or after the interview. The interviewees all agreed to being recorded and signed an agreement sharing their answers within the context of this research, and, to provide further face validity, each of them was granted the opportunity to review the answers.

### Instruments

For the standardized open-ended interview, participants were approached by phone and, or by mail with requests for their participation and detailed explanation of the purpose of the research interview and their rights as a participant.

The interviews lasted approximately one hour, three were conducted in person, in the respective office of each participant, and three were conducted by means of a web conferencing tool. The interview template contained a small number of closed and open-ended questions relating to online problem-based, case-based and competency-based (or oriented) learning activities and curricula.

The departing point for the interview questions with regard to this research are the competencies relating to health promotion professionals. These were adopted from the Core Competencies Framework for Health Promotion Handbook, which defines competencies of health professionals as “a combination of the essential knowledge, abilities, skills, and values necessary for the practice of health promotion” (Barry et al. 2012, p. 7). The core knowledge constitutes the main competencies, “health promotion knowledge” and “ethical value” surrounded by skills and abilities such as: “advocate for health”, “enable change”, “mediate through partnership”, “communication”, “leadership”, “assessment”, “planning”, “implementation”, and “evaluation and research”, that are divided into sub-competencies (Barry et al. 2012, pp. 10–18).

The interview was started by ensuring that the interviewee was aware of the purpose of the interview and key-terminology by going over written definitions of the key-terminology, namely: ‘online learning’, ‘problem-based learning’, “case-based learning” and ‘competency-based learning’, followed by the interviewer reading the questions of the and elaborating if required.

Interview question one through three addressed the characteristics of their student population, the online program(s) the participant represents. Question four and its’ sub-questions try to determine whether the program followed a competency-based model and if so which competencies their program(s) aim(s) to develop. This was designed as a set of dichotomous questions (yes/no) questions asking specifically for the competencies from the Core Competencies Framework for Health Promotion Handbook, in order to best serve the program director of Maastricht University Master Health Education and Promotion (HEP), followed by the possibility to add additional competencies that are not necessarily part of the framework. The inclusion of the dichotomous questions served funnel toward open-ended follow up questions (Cohen et al. 2000, p. 250).

Question four and its’ subset of questions asked to name and describe the most effective course activities in developing (about 2 to 5) competencies (learning outcomes), and to explain for each activity if they are problem/case-based, if and how they relate to constructive, collaborative, contextual, or self-directed learning, and if and how the relate to competency development and assessment and to what extend a student could determine their own pace. The fifth and final question relates to online communication. To ensure construct validity, the chair of a distance education master’s program reviewed the interview questions. See appendix for full interview questions and definitions.

The second instrument, the literature review, covered the fields of education, distance and online education, health sciences and medicine, addressing theory, current research and past research, and (best) practices within the field, sourced from EBSCO, LearnTechLib, ERIC, Education Research Complete, PubMed, and Medline in 2018. The reviewed literature covered peer reviewed journal articles and books published between 2000 and 2018, written in both English and Dutch. Search words included combinations of the following search terms Health sciences, online, online education, online learning, online learning activities, competency-based education, competency-based education, competency-oriented education, competency-oriented learning, case-based education, case-based learning, problem-based learning.

### Data analysis

The interviewer and author of this paper recorded and transcribed the interviewee responses. Where the interview was conducted in Dutch, responses have been translated into English. In various instances, additional comments relevant to this research have been included in the interview answers. The interviewees then reviewed the interview questions and could use the opportunity to change or add information to clarify their answers.

Finally, a content analyses of the open-ended questions to supplement the findings from the literature review. The analyses included counting the frequencies of certain responses and practices, followed by clustering into categories practices and classifications (Cohen et al. 2000).

The literature review did not produce English or Dutch literature on merging online problem-based or case-based learning with and online competency-based learning approach. It provided limited comparisons of mentioned learning approaches, and the author did not come across literature that identified learning activities that are suitable for merging online competency-based education with either problem-based or case-based learning. Reviewed literature did contain description of online course design of both problem-based and case-base learning, as well as curriculum design and learning activities for competence-based learning. A comparative analysis was conducted of literature on each individual learning approach to identify common factors, as well as challenges and opportunities for merging online competency-based education with online problem- and/or case-based learning.

## Results

### Constructing a competency-based curriculum

Even though this research focuses on learning activities, it is difficult to look at them as isolated items, particularly in competency-based education, as the design starts at the finishing line, i.e., the outcome or competency and then moves backward to each activity. In other words, competency-based education first asks what the learner should be able to do at the end of a program and then maps backward (Albanese et al. 2010; Frank et al. 2010; Gruppen et al. 2012; Porter & Reilly, 2014). For each competency, a detailed benchmark is provided that specifies what type of proof a learner needs to present to confirm competency attainment (Gruppen et al. 2012).

Planning a competency-based (medical) curriculum in six steps.

1. Identify the abilities needed of graduates.

2. Explicitly define the required competencies and their components.

3. Define milestones along a development path for the competencies.

4. Select educational activities, experiences, and instructional methods.

5. Select assessment tools to measure progress along the milestones.

6. Design an outcomes evaluation of the program.” (Frank et al. 2010).

In actuality, the majority of final competencies cannot be learned at once; it takes a step-by-step process. Albanese et al. suggest that the broken down levels of each competency should be referred to as “progress competencies, pre-competencies, sub-competencies or proto-competencies” (2010, p. 441). The time-span does not necessarily equal the length of a course module or curriculum. Moreover, both learning and assessment of learning may include both in-class and out-of-class activities.

Mapping a curriculum based on learning outcomes to one based on competencies can be challenging. The MDPharm program in Poland reviewed the learning outcomes of the program, divided them into “knowledge,” “professional skills” and “social skills” and compared them with the competencies of the European Competency Framework to identify the gaps (Skowron et al. 2017, pp. 2–3). In the US, various universities have implemented competency-based education, many of which are online universities, as competency-based education may appeal to the same target student group of working professionals who seek a flexible learning approach (Porter and Reilly 2014).

Only one of the interviewed participants, who runs a program geared to working professionals, has built a competency-based curriculum. The participant provided the following example of mapping backwards:

In our course PALC 615, Advanced Pain Management, Module 2 we have a weekly module objective. An example is as follows: give an actual or simulated patient with a complaint of pain, convert between dosage formulations and routes of administration for the same opioid (e.g., morphine, hydrocodone).

The learning activities that enable the student to achieve this module objective include activity 1, required reading; activity 2, CAPC Course 9.

This supports the following course objective: convert an actual or simulated patient from one opioid regimen to a different opioid regimen, and provide an explanation of your rationale.

This supports the terminal performance objective: contribute as part of the interdisciplinary team in the assessment and management of pain and/or other physical symptoms that demonstrate evidence-based best practices.

The other five participants reported that their programs defined competencies without departing from the time-based curriculum structure. This low number corresponds with the lack of literature on online competency-based curriculum design and learning activities in higher education. Interviews that other researchers conducted with representatives of competency-based university programs reveal that the implementation of a competency-based curriculum frequently involves a reorganization of faculty into two groups: teaching faculty and full-time student coaches or mentors (Porter and Reilly 2014).

### Building and assessing competencies in an online case- or problem-based learning environment

Constructing an online environment that facilitates building and assessing competencies online can be a complex task, particularly those steps in competence development that involve action and experience, and the last steps toward professional proficiency, are challenging to support through an online environment. According to Ehlers, Scheckenberg and Adelsberger, an online environment that affords competence development provides the learner with the opportunity to internalize their learning by performing in circumstances that vary in terms of complexity and certainty (2008). They suggest that a “problem-oriented, authentic, collaborative” online learning environment would best support the needs of a competency-based approach. Current online learning environments and digital tools afford interaction (between teacher and student as well as among students), collaboration, feedback, reflection, and synchronous communication, making them increasingly suitable for competency development (Ehlers et al. 2008), as well as for problem- and case-based learning scenarios*.* Nevertheless, certain actions or experiences may or cannot take place online. In that case, the student may perform the skill in a real setting (i.e., work placement), followed by online reflection, for instance, through a discussion post followed by peer feedback, or adding a reflection to an e-portfolio (Ehlers et al. 2008).

Assessment is central to competency-based education; a demonstration of an acquired competency awards the student with credit and/or allows the learner to move on toward a next competency level, blurring the lines between learning and assessment. A frequently used and suitable instrument for competency assessment is a portfolio, in which learners present proof of their acquired competency. For valid assessment, it important that the student presents authentic, valid, and recent proof that he or she possesses a competency at the desired level (Van Berkel et al. 2014). A second instrument is a competency-based interview conducted by preferably more than one assessor. Van Berkel, Bax and Joosten-Ten Brinke, recommend the “STARTT-method”, which follows the following structure for the interview: situation, task, action, result, reflection and transfer” (2014, p. 104). A third assessment instrument is a professional artifact, i.e., a product manufactured by or skill performed by a student, such as an analyses, an advice, a visual or schematic design of a product or an intervention (Van Berkel et al. 2014). Whereas the first three types of artifacts can be easily created and submitted in an online environment, an online intervention may be more challenging. Innovative, affordable video and audio recording or streaming possibilities, combined with (video) feedback tools, yield skill demonstration as well as remote feedback among students or from a teacher. One participant, who runs the palliative care program, provided an example of video assessment:

I ask my palliative care students to have themselves video recorded performing a certain skill and submit the recording. I will then assess the student performance and provide feedback, combining learning and assessment.

### Online competency building, learning and assessment activities in problem-based and case-based programs

#### Simulations, role-play and serious games

Various online platforms offer virtual case-based learning activities, using either virtual or (anonymized) real cases. Examples of such platforms are the Extension for Community Healthcare Outcomes (ECHO), and Cases for teaching and Learning (CASTLE). On the latter, the course developer or tutor can author different case scenarios (Ali et al. 2018). One of the participants reported on a virtual patients learning program that was used by several medical programs. This platform provides dozens of virtual patient cases for medical students to practice on. This facilitates the development of medical reasoning skills in these students.

Another example of an online case-based training program for healthcare learning is “Patient Assessment Training System PATSy”. This is a repository of records virtual, and various uncommon and real (anonymized) patient cases. These platforms typically combine videos, assessments, and patient histories (Ali et al. 2018; Cox 2011), which learners can practice “diagnostic reasoning” and “clinical skills”. If required, students can repeat the same patient case as many times as needed (Cox 2011, p. 41), making it a suitable tool for both case-based and competency-based education. Ali et al. signaled the lack and need for tutor feedback in this platform, which is an important component of both case- and problem-based learning. They compared it to a platform called interactive case-based learning system (iCBLS) in which the tutor can interact with the students for guidance, assessment and feedback, rather than the automated feedback that seems common in mentioned platforms (2018). In addition, it is equipped with a timer, which aids in determining the complexity of the next case. These features would facilitate a competency-based model as it affords the assessment of a learner throughout the learning process and provides the students the opportunity to improve.

One of the interview participants is the director of an online graduate palliative care program. Her students include physicians, nurses, pharmacists, social workers, and chaplains, evenly divided over small collaborative teams working on cases. This approach is authentic, self-directed, and personalized. The interviewee explained:

Cases presented to students, combine a description, a patient history, online videos of staff, and actors role-playing scenarios. The case scenarios are increasingly complicated to scaffold student skills development.

Students are very happy; they say it is very practical and applied and very pertinent to their learning needs.

Creating multi-disciplinary teams with students with various professional backgrounds and areas of expertise, for instance a physician, pharmacist, social worker, nurse, and a chaplain. This facilitates knowledge ‘cross-pollination’ and building skills needed to work across disciplines in real-life situations.

Role-play is a learning activity that is characteristic for experiential learning approaches such as PBL and CBL; it can simulate authentic, complex situations and promote problem-solving skills (Hou, as cited in Ching 2014). Ching reports on a case-based role-play scenario where students play stakeholder roles in VoiceThread, a platform that features video, audio, text sharing and commenting (2014). Such a role-play scenario affords competency development and assessment. Roles and scenarios facilitate competencies such as communication, advocating, mediation, but also planning and research and for developing leadership skills. De Nooijer describes a virtual environment world, similar to second life, developed for health science students to implement a health prevention measure for prevention of post-natal depression in women at risk (2013). One of the interview participants worked the virtual environment described by de Nooijer. The participant reported:

In the virtual environment, students make appointments with stakeholders, such as insurance companies, nurses, government officials. The goal is to advance the implementation of the prevention measure. The virtual environment provides authentic problem solving learning activities that facilitate both practice and assessment of many important health promotion professional competencies. The weakness of our virtual environment was that real faculty played the stakeholders. This makes interaction very real, but teaching staff do not always have time, so the number of times a student can repeat a task is limited.

#### Online collaborative groupwork

Another learning activity that fits both a case- and problem-based learning approach is collaborative groupwork. One participant details how health education and promotion master students asked to design an intervention mapping protocol collaboratively. Students are encouraged to select their own problem case, promoting authentic, self-directed, personalized learning. All participants who use collaborative group work leave it up to the students to select their preferred mode of communication. For online brainstorm sessions (part of the PBL process), students often communicate synchronously. To facilitate a more effective group process and curb so-called ‘free-riders’, students are asked to document their agreements and discussion and rotate roles. As one interviewee explains:

How the subgroups communicate is by choice, but the tutor does want to be able to monitor communication. The only requirement is that brainstorm needs to be documented. Sometimes, they communicate synchronously and sometimes asynchronously. Students usually agree on task division and method of contact. Their brainstorm sessions are usually synchronous and recorded in google docs. Most pick skype for video conferencing and then report through the group discussion area of elevate (the online course environment).

The group size is about four students. They have to pick a different leader per week. The leader initiates the process and determines deadlines. Each group needs to hand in the document that records the group process as an assignment.

Access to online group work provides teachers the opportunity to guide and assess both quantity and quality of the group discussion, the collaborative process, and student competency development.

#### Peer feedback and reflection

In case-based and problem-based learning, peer feedback is an important reflective learning and assessment activity that facilitates constructive learning process of making sense of, reflecting upon, and expressing their own reasoning to peers (Ching 2014). Practicing personal and interpersonal competencies and skills, such as critical thinking, assessing, evaluating, and communicating, may be applied to “formative and summative assessment”; research reports more positive impact when using the former (Ching 2014). Facilitating and encouraging peer feedback in an online environment may also enhance student interaction and engagement. Peer feedback can be provided in writing, as an audio recording or as a video. This facilitates either writing or presentation skills as well as the opportunity for the tutor to assess those skills. Four participants reported on applying peer feedback and one assesses students on the feedback they provide.

In order to promote reflective learning, four participants ask their students to search literature, relevant to their project, problem, case, or learning gap, and write an annotated bibliography or an essay connecting theory to practice. This caters to both (academic) skills and knowledge development in students and facilitates meaningful assessment. One participant describes that reflect on the learning and collaborative process itself:

Students are asked to make a group reflection and an individual reflection. The individual reflection addresses collaboration and division of tasks and on the students’ own functioning and tasks. The group reflections discusses on what went well and what did not go well. How were those issues dealt with?

#### Synchronous and asynchronous online discussion

Discussion is a central element of problem-based learning and an excellent way to build interpersonal competencies for constructive learning, reflection, and analyses. When taking place online, discussion group members are separated in space and possibly in time. With current state of audio and video conferencing technology, it is possible to bridge distance gap, while adhering to a synchronous discussion approach that resembles face-to-face-interaction.

Verstegen et al. assert that synchronous communication through web conferencing tools can be as effective as face-to-face group discussion, provided it is well prepared (2016). One interview participant, a blended and online learning research coordinator, who makes frequent use of synchronous communication in a hybrid format, explains the format and confirms the need for thorough preparation:

We communicate synchronously via a web conferencing platform and an interactive screen. This combines a physical classroom where teacher, moderator, and students are present and connected with about 24 remote students who participate in class via large screens in the back of the room, equipped with speakers and microphones. Engagement is a considerable challenge, as remote students need more encouragement than on-campus students do. Techniques, such as polls, promote remote student engagement. The teacher needs an extra moderator to facilitate interaction and engagement, and provide technical support.

Two participants organize discussion hours with visiting experts. As one participant who runs the palliative care program explains:

We offer an expert-led themed discussion that is optional. Usually ten to thirty students out of our groups of about forty students log in. Students who could not attend can watch the video.

This caters to flexibility of part-time and/or international students who often mix a professional career, in the case of health science students with odd working hours. Four interview participants, however, indicate that synchronous discussion is complicated to organize when catering to students who juggle many responsibilities, such as professional careers and family lives, and who live in different time zones. As one participant who runs an international health education program explains:

Communication is largely asynchronous, because our students come from all over the world and many work irregular shifts, as they are health professionals.

At the same time, having students from different disciplines, backgrounds, and cultures, promotes their ability to work in interdisciplinary and intercultural teams, which are precisely the type of competencies a health science student needs to develop.

The use of asynchronous communication methods, in lieu of synchronous communication, can address this challenge while adhering to the constructivist and learner-centered approach that form the core principles of problem-based and case-based learning as well as competency-based education. In comparative studies of online and face-to-face case-based learning, the observations suggest that learning outcomes of online PBL are slightly more positive, although the difference was not significant (Moeller et al. and Raupach et al., as cited in Nicklen et al. 2016). A comparative study of online and face-to-face CBL by Nicklen et al. suggested that benefits of online learning are student flexibility, while the subjects of their research reported that technology and communication were important, but challenging aspects of online case-based group discussion (2016).

Three participants combine asynchronous communication with (self-)reflection activities by asking students to write a reflective post and react to posts by others. Although interaction is less spontaneous, such posts facilitate constructive learning and deeper reflection, and held develop writing competencies (Hawkes as cited in Verstegen et al. 2016), as it forces the learner to formulate their thoughts more carefully. It also provides the teacher an opportunity to assess both content and mentioned competencies. One participant reports on organizing group discussions within a synchronous platform that teachers can break into for providing guidance or assessment.

### Challenges

#### Costs and logistics

The implementation of competency-based education involves an exhaustive redesign of the entire curriculum, assessment, and faculty support. Mentoring activities replace teaching activities to a considerable extent, drastically changing the roles of faculty, and the administrative system, which usually follows a time-based curriculum (Porter and Reilly 2014). Per contra, Porter and Reilly (2014) suggest that competency-based education may even lead to cost reduction for students and institutes. Particularly, mid-career students could save money, as competency-based programs provide them with the opportunity to demonstrate previously acquired competencies and receive credit, i.e., exemption. Several US higher education professionals interviewed by Porter and Reilly (2014), indicated that once the investments for curriculum and system overhaul are completed, a competency-based curriculum could be scaled up for cost reduction.

One of the interview participants indicated that the limits of the institutions LMS and digital feedback tools were a strong limiting factor. This participant, who runs a master in health professions education, stated:

Blackboard, our current LMS has a linear design. It does not allow for pairing two students for a feedback exercise upon completion of a task, but forces us to use a preset assignment deadline. This makes self-paced assessment, impossible. In two years, we want to redesign our program into a more competency-based format. The current LMS may present serious challenges. I hope that the LMS our university will migrate to, will have a more fluid environment. If not, we will be considering a website coupled with digital tools.

#### Educational legislation and accreditation

Institutes and research articles that discuss the implementation of competency-based education, report on their focus on outcomes without necessarily parting with their time-based curriculum planning, quoting accreditation or other bureaucratic constraints (Barman et al. 2014). Skowron et al. call for a change of the cultural mindset that enables a factual move from learning objectives to competency-based education (2017).

In Europe, the 1999 Bologna declaration and following Bologna process, aim to promote learner mobility. To facilitate recognition of learning and study credits the European Credit Transfer System (ECTS) was introduced (Van Berkel et al. 2014). The design of the ECTS system is originally tied to traditional, time-based recognition of credits, certificates, and diplomas, and designed around intended learning outcomes.

Around the start of the millennium, the Dutch education system introduced a system to recognize acquired competencies (EVC), mainly aimed at vocational training and universities of applied sciences. (Van Berkel et al. 2014). In the US and Europe, accreditation bodies for higher education institutes are usually supportive of the introduction of competency-based curricula and assessment (Porter and Reilly 2014).

## Discussion

Competency-based education has gained popularity in education and workplace training; yet, no single agreed upon definition appears to exist (Schnoepner-Torres et al. 2015). Meanwhile, Problem-based learning has a thirty-year history (Savery 2015) and numerous articles defining and describing the approach. At institutions where PBL is practiced, not all teaching staff members seem fully familiar with the theoretical underpinnings. Even at an institute as Maastricht University, where the large majority of teaching staff has been instructed on PBL, many tutors would feel uneasy to step away from the (seven-step) procedure (Moust et al. 2013), rethink the core values of problem-based learning and come up with online activities. What really are differences and similarities with case-based learning? How can we integrate problem- and case-based learning with competency-based education in an online environment?

The purpose of this research is to identify suitable practices, as well as challenges, for online course design and online learning activities for higher education health science programs, when integrating a competency-based education with an online problem-based, case-based learning approach. Furthermore, it attempts to bring clarity to the professional conversation by defining and analyzing online problem-based, case-based learning, and competency-based education, and to inform and inspire faculty and staff in the field of health sciences, involved in delivering or supporting a mixture of mentioned education approaches in an online environment.

This paper has searched and identified common characteristics of problem- and case-based learning, and competency-based education. These include student-centeredness, self-directedness, skill building and a constructivist, authentic, experiential approach to learning. In case of health sciences, skills and competencies include higher order thinking skills and problem-solving skills (Tan et al. 2018; Ehlers et al. 2008).

Interview responses produced a considerable number of learning activities suitable for case-based and problem-based learning, as well as competency-based education. One participant complained how the rigid characteristics of their institute’s LMS required many workarounds to facilitate cohorts of students following individual learning paths and building individual competencies online. Workarounds that required considerable effort from administrative and teaching staff for monitoring and supporting students.

Only one of the participants was actually involved in a mixed case-based and competency-based curriculum, determining terminal performance objectives (TPOs) first, and then mapping backwards through various learning activities. The other participants could define competencies student would develop during a given program, but had not departed from a time-based curriculum. Hence, their curriculum is best defined as competency-oriented, as it provided limited opportunity for individually paced learning and assessment. All participants confirmed claims found in reviewed literature that, one of the greatest challenges for transforming a time-based curriculum, either problem-based or case-based, into a competency-based curriculum relates to logistical modifications and administrative support requirements posed by a personal learning path for each learner. Such a learning path requires competency assessment opportunities at any given moment, and extra time for practice and/or personal instruction (Scheopner-Torres et al. 2015). This means assessors need to be available to do individual assessments and likewise, mentors, specialists or teaching staff need to be available for instruction on demand. Problem-based learning and case-based learning, however, generally require extensive collaboration and reflection among learners. When learners go through the curriculum at the same pace, it is easier to organize collaborative group work and discussion that are relevant to all learners, then when learners can move at their own pace.

Learning activities identified by participants, include increasingly difficult case scenarios to scaffold student skills development for diverse groups of adult learners. Although such activities promote the development of various key competencies, it does not allow students to stop and ask for assessment at any given time. An e-portfolio in which a student share an artifact that is proof of his or her skill coupled with a reflection on his or her own learning could address this challenge. Role-play scenarios in virtual platforms seem to provide greater opportunity for assessment any time, as well as many practice runs. However, such a virtual platform does not necessarily simulate collaboration. It seems merging online competence-based education with online problem- or case-based learning, requires compromise. Nevertheless, if carefully structured, a curriculum that merges online CBE with PBL and CBL can incorporate most elements of all education approaches.

### Limitations

The author notes that the availability of research publications on online competency-based education has proven scarce; a finding confirmed by multiple authors of other research publications on (online) competency-based education (Porter and Reilly 2014; Tan et al. 2018). In addition, the number of participants of this study was too limited to provide a full overview of the current situation at higher education health science programs merging online problem- or cased-based learning with online competency-based education. The author attributes this limited number of participants to time limitations and to the fact that, European higher education institutions were only exploring a switch to an online competency-based curriculum.

## Conclusion

Merging a problem- or case-based learning approach with competency-based education faces a number opportunities and challenges. As problem- and case-based learning put students in the center and focus on competency and skills development, rather than content knowledge, these learning approaches appear to be suitable for merging with competency-based education, which also focuses on skill and competency development. Moreover, PBL/CBL and CBE all aim to enhance transferable knowledge skills and turn their students into life-long learners (Blumhof et al. 2001; Cheaney and Ingebritsen 2006). Problem- and case-based learning generally have a collaborative character, and include group discussion, brainstorming, group product delivery and peer feedback. All are aimed at enhancing, deeper more meaningful learning, but also at the development of interpersonal skills, communicative skills, (self-) reflective skills, leadership skills, all pivotal for success in a twenty-first century workplace. At first sight, the collaborative character of problem-based and case-based learning seems at odds with the personalized learning approach of competence-based education. Nevertheless, skills such as leadership and communication can be developed most effectively in a collaborative environment. Competency-based education should thus offer the possibility to participate in group work. Reviewed literature and interviewed participants identified various online activities that facilitate building and assessing competencies online. The identified activities include peer feedback, role-play, online collaborative group work, virtual case-based activities, online discussions, and reflection. Additional assessment strategies include the creation of an e-portfolio or asking a student to film themselves performing a skill. At the same time, an (online) competency-based curriculum should provide the student with the opportunity to demonstrate a skill at any given moment during the program, rather than on a predetermined deadline.

Merging competency-based education with PBL and CBL, seemingly offers various opportunities in terms of learning activities. Challenges are high start-up costs and the consequences of changing the logistics of a program and faculty support roles to facilitate a personal, self-paced learning path, (Porter and Reilly 2014). The final challenge, suggested by an interview participant, involves finding an LMS that is fluid enough to support this flexibility.

The support of competency-based curricula development by accreditation bodies and employers potentially promote a more widespread implementation of competency-based curricula, particularly in health sciences. In addition, Covid-19 provoked a sudden transition to online education. In order to support these trends, the author recommends further study to support teaching staff and program coordinators and directors who intend to merge case- or problem-based learning with competency-based education in an online learning environment.

## Electronic supplementary material

Below is the link to the electronic supplementary material.

Electronic supplementary material 1 (DOCX 21 kb)

## Data Availability

The transcripts of the interviews are available upon request, and can be obtained by sending the author of this paper a request by email (see author contact information). Participants responses provided in interviews conducted in Dutch have been translated into English.
