# Supplementary material for: Integrating competency-based education with a case-based or problem-based learning approach in online health sciences
Source: Asia Pac Educ Rev. 2020 Nov 18;21(4):683–96. doi: 10.1007/s12564-020-09658-6 (PMC7672406; doi:10.1007/s12564-020-09658-6)
Supplement: Supplementary file 1 — Electronic supplementary material 1 (DOCX 21 kb) [file 12564_2020_9658_MOESM1_ESM.docx]

**Interview Questions**

**Online Learning Activities**

# **Intro**

Thank you in advance for taking the time for an interview. You are receiving this document with interview questions, for your information. You do not have to answer any questions in advance. The interview will be conducted in person or via phone or skype.

With your permission, the interview will be recorded to facilitate accurate reproduction of the answers. You, the interviewee, reserve the right to request confidentiality and the omission of any (part) of their responses, during or after the interview. An agreement will co-signed by the interviewee and the interviewer (researcher). After the interview, you will get the opportunity to review the interview notes.

# **Start Interview**

Your Name:

Institute:

Department/faculty/program:

1. What blended or online courses/programs are you involved in (study year/topic/credits)?
2. What is your role?
3. Can you give the general characteristics of the students (age/previous experience/ professional? Full time students – located where?

**Competence-Based Learning**

1. Does the program

A Follow a competence based-model?

B Work toward building competencies?

- 1. If yes, please indicate which competency is addressed and how?
     - Ethical values yes/no
     - Health Science knowledge yes/no
     - Enable Change yes/no
     - Advocate (for health) yes/no
     - Mediate yes/no
     - Communicate yes/no
     - Leadership yes/no
     - Assessment yes/no
     - Planning yes/no
     - Implementation yes/no
     - Evaluation and research yes/no
     - Different competencies, namely
       - X………………………….
       - Y……………………………
       - Z…………………………..
       - Etc…

**Problem- or Case-Based learning**

1. Name and describe the most effective course activities in developing (about 2 to 5) competencies (learning outcomes) & explain for each activity if they are problem/case-based & if/how they relate to:
   1. Constructive/ Collaborative/Contextual/Self-directed learning
   2. Developing one or more competencies
      - Which competencies
      - How addressed?
      - How assessed?
      - Does it offer the student the opportunity to repeat or build a skill or competence at his/her pace until the competence is acquired?
   3. How does the communication take place both student-teacher and/or student-student (i.e. asynchronous or synchronous, group size)?
      - Could you name challenges and benefits?

# **End Interview**

# **Survey supplement - Key terminology**

**Online learning** is learning that uses computer networks, such as internet, intranet and online learning networks as a medium for synchronous or asynchronous interaction, instructor facilitated, or exclusively computer based (Harasim, 2012).

**Problem-based learning** is an experiential, student-centered approach to learning that facilitates the integration of theory and practice, and application of knowledge and skills in order to come up with to a viable solution to an ill-defined defined, real-world (authentic) problem; problem-based learning is a self-directed, constructive and collaborative process (Savery, 2015).

**Case-based learning** is an experiential, self-regulating approach to learning aimed at activating learners and promoting higher order thinking skills, i.e. analyses and synthesis, and critical thinking skills, problem-solving skills and self-reflection, while working through a well-constructed case. The aim is to build knowledge that can be applied to similar situations in the future. If conducted as a group, a case study stimulates collaboration (Savery, 2015; Lyons & Bandura, 2017).

**Competency-based learning** relates to “reforms in which students demonstrate mastery of a defined set of competencies in lieu of completing credit requirements based on time in class” (Schnoepner-Torres, Brett & Cox, 2015, p1.); once mastery is demonstrated through different forms of assessment, often involving the application of knowledge, the learner can advance. If mastery is not achieved, the learner is allowed more opportunity to learn. Learning can take place in a face-to face or online classroom setting, as well as outside the classroom; the assessment often involves feedback by peers as well as self-assessment.

**Health Sciences** can be defined as all sciences relating to health care such as health education and promotion, disease prevention, and policy management and evaluation of health.
